# Antileishmanial Activity of Flavones-Rich Fraction From *Arrabidaea chica* Verlot (Bignoniaceae)

**DOI:** 10.3389/fphar.2021.703985

**Published:** 2021-07-20

**Authors:** João Victor Silva-Silva, Carla Junqueira Moragas-Tellis, Maria do Socorro dos Santos Chagas, Paulo Victor Ramos de Souza, Celeste da Silva Freitas de Souza, Daiana de Jesus Hardoim, Noemi Nosomi Taniwaki, Davyson de Lima Moreira, Maria Dutra Behrens, Kátia da Silva Calabrese, Fernando Almeida-Souza

**Affiliations:** ^1^ Laboratory of Immunomodulation and Protozoology, Oswaldo Cruz Institute, Oswaldo Cruz Foundation, Rio de Janeiro, Brazil; ^2^ Laboratory of Natural Products for Public Health, Pharmaceutical Techonology Institute – Farmanguinhos, Oswaldo Cruz Foundation, Rio de Janeiro, Brazil; ^3^ Student on Postgraduate Program in Translational Research in Drugs and Medicines, Farmanguinhos, Oswaldo Cruz Foundation, Rio de Janeiro, Brazil; ^4^ Electron Microscopy Nucleus, Adolfo Lutz Institute, São Paulo, Brazil; ^5^ Postgraduate in Animal Science, State University of Maranhão, São Luís, Brazil

**Keywords:** *L. amazonensis*, flavonoids, *Arrabidaea chica*, macrophages, nitric oxide, transmission electron microscopy

## Abstract

Acknowledging the need of identifying new compounds for the treatment of leishmaniasis, this study aimed to evaluate, from *in vitro* trials, the activity of flavones from *Arrabidaea chica* against *L. amazonensis*. The chromatographic profiles of the hydroethanolic extract and a flavone-rich fraction (ACFF) from *A. chica* were determined by high-performance liquid chromatography coupled with a diode-array UV-Vis detector (HPLC-DAD-UV) and electrospray ionization mass spectrometry in tandem (LC-ESI-MS-MS). The flavones luteolin (**1**) and apigenin (**2**), isolated from chromatographic techniques and identified by Nuclear Magnetic Resonance of ^1^H and ^13^C, were also quantified in ACFF, showing 190.7 mg/g and apigenin 12.4 mg/g, respectively. The other flavones were identified by comparing their spectroscopic data with those of the literature. The *in vitro* activity was assayed against promastigotes and intramacrophagic amastigote forms of *L. amazonensis*. Cytotoxicity tests were performed with peritoneal macrophages of BALB/c mice. Nitrite quantification was performed with Griess reagent. Ultrastructural investigations were obtained by transmission electron microscopy. Anti-*Leishmania* assays indicated that the IC_50_ values for ACFF, apigenin, and luteolin were obtained at 40.42 ± 0.10 and 31.51 ± 1.13 μg/mL against promastigotes, respectively. ACFF and luteolin have concentration-dependent cytotoxicity. ACFF and luteolin also inhibited the intra-macrophagic parasite (IC_50_ 3.575 ± 1.13 and 11.78 ± 1.24 μg/mL, respectively), with a selectivity index of 11.44 for ACFF. Promastigotes exposed to ACFF and luteolin exhibited ultrastructural changes, such as intense cytoplasm vacuolization and mitochondrial swelling. These findings data evidence the antileishmanial action of flavone-rich fractions of *A. chica* against *L. amazonensis*, encouraging further studies.

## Introduction

Flavonoids are an important class of secondary metabolites with a low molecular weight polyphenolic structure, widely distributed in the plant kingdom among subgroups that include chalcones, flavones, flavonols, and isoflavones (Panche et al., 2016). This metabolic class has its biological and therapeutic activity experimentally determined (Nijveldt et al., 2001), being able to affect enzymes and various cellular systems, having beneficial effects on the body (Silva et al., 2010). Furthermore, it is largely known that flavonoids have a wide spectrum of antileishmanial activity (Fotie, 2008; Wong et al., 2012; Rocha et al., 2018).

Leishmaniasis is a neglected tropical disease that seriously affects humans and can lead to death if left untreated (Reithinger and Dujardin, 2007). This protozoosis represents a global health challenge, since it has a worldwide distribution, with an estimate of more than one billion people living in endemic areas and at risk of *Leishmania* infection (World Health Organization, 2020). In addition to these circumstances, the anti-leishmanial drugs currently in use exhibit drug resistance, toxicity, and high cost, which may explain the low adherence to treatment (Sundar et al., 2019). The lack of new therapeutic alternatives to leishmaniasis highlights the need to seek new compounds with leishmanicidal activities. In this context, the use of natural products in traditional medicine has contributed to the identification of candidate compounds for the development of new drugs. Therefore, medicinal plants represent a repository of bioactive compounds potentially useful for the development of new therapeutic alternatives for leishmaniasis (da Silva et al., 2018).

*Arrabidaea chica* (Humb. & Bonpl.) B. Verlot, syn. *Bignonia chica*, belongs to the family Bignoniaceae. It comprises about 120 genera and 860 species (Fischer et al., 2004; Sampaio et al., 2016). *A. chica* occurs in tropical America, being a very common species in the Amazon region (Takemura et al., 1995; dos Santos et al., 2013) and is popularly known as crajiru or pariri (Behrens et al., 2012). It is traditionally used as a medicinal plant in the Amazon region (Takemura et al., 1995), with the use of tea made from leaves as an anti-inflammatory (Evangelista et al., 2013), to treat skin inflammation and mycoses (Corrêa, 1984), and has astringent properties (Lima de Medeiros et al., 2011). Amazonian Indians use the decoction of leaves to clean wounds and ulcers to aid in healing, in addition to the use to treat fungal infections and herpes (Lorenzi and Matos, 2002), as well as for other skin conditions (Barbosa et al., 2008). Furthermore, the infusion (oral use) is used to heal wounds and cleanse the blood (Bieski et al., 2012). Previous studies have demonstrated its antioxidant (do Amaral et al., 2012; dos Santos et al., 2013), wound healing (Aro et al., 2013; Cortez de Sá et al., 2015), trypanocidal (Barbosa et al., 2008; Miranda et al., 2017) and leishmanicidal activities (Rodrigues et al., 2014; Cortez de Sá et al., 2015; Moragas-Tellis et al., 2020). In the phytochemical screening of extracts of *A. chica*, the leaves are rich in anthocyanidins, such as carajurin and carajurone (Moragas-Tellis et al., 2020); in addition to some flavones (Paula et al., 2013), such as luteolin and apigenin (do Amaral et al., 2012).

Thus, the present study aimed to evaluate the antileishmanial activity *in vitro* of the hydroethanolic extract derived from *A. chica*, as well as of its flavone-rich fraction and the isolated flavones, luteolin and apigenin, against promastigotes, and intracellular amastigotes of *Leishmania amazonensis*.

## Materials and Methods

### Plant Material

Leaves of *A. chica* Verlot (Bignoniaceae) (morphotype IV) were cultivated and collected in March 2016 at Fiocruz Atlantic Forest Campus, municipality of Rio de Janeiro, Rio de Janeiro State, Brazil (S22.9406 W43.4046). Plant material was identified by Dr. Marcus Felipe Oliveira da Silva at the Botanical Collection of Medicinal Plants of Farmanguinhos/FIOCRUZ, where a voucher specimen was deposited and registered under the number CBPM666.

### Extraction and Isolation

*Arrabidaea chica* leaves were dried in a forced circulation oven at a temperature of 45°C for 4 days. After drying, the plant material was powered using a knife-mill affording 0.85 mm particles that were stored in an amber flask. The hydroethanolic extract was prepared by exhaustive maceration of dried and powdered leaves (1.5 kg) in 70% ethanol solution (v/v) with three changes of solvent, once every 48 h for 7 days at room temperature. After filtration, the solvent was evaporated under reduced pressure to yield 226.19 g of a red extract (ACCE) corresponding to yielding of 15.07%. The crude hydroethanolic extract (60 g) was then submitted to liquid-liquid partition with *n*-hexane (3 × 200 mL), dichloromethane (3 × 200 mL), ethyl acetate (3 × 200 mL) and *n*-butanol (3 × 200 mL). The final aqueous residue was discarded. Dichloromethane fraction was successively chromatographed by column chromatography on Sephadex LH-20 (Sigma, St Louis, MO, United States), using methanol as eluent to produce a purified flavone-rich fraction determined by thin layer chromatoghapy (TLC) analysis, and named ACFF. Successive chromatographic fractionation steps of ACFF on Sephadex LH-20 led to the isolation of two flavonoids (F1 = 24 mg; F2 = 4 mg). Isolated compounds and a flavone-rich fraction were analyzed by TLC (silica gel F_254_, Merck, Darmstadt, Germany) using acetone: chloroform: formic acid (75:16:0.8 v/v/v) as eluent and, subsequently, sprayed with 1% NP/PEG reagent (diphenylboriloxyethilamine/polyetileneglicol, Sigma, St Louis, MO, United States), as well as ^1^H and ^13^C NMR spectrometry. Comparison with literature records allowed the identification of **(1)** luteolin and **(2)** apigenin (Ersöz et al., 2002; Özgen et al., 2011; Siraichi et al., 2013; Grabsk et al., 2017).

**(1)** Luteolin 1H NMR (400 MHz-methanol-d6) d: 6.47 (s, 1H, H-3); 6.13 (d, 1H, H-6, J = 2.0 Hz); 6.35 (d, 1H, H-8, J = 2.0 Hz); 7.35 (d, 1H, H-2′, J = 2.2 Hz); 6.88 (d, 1H, H-5′, J = 8.2 Hz); 7.35 (dd, 1H, H-6′, J = 8.2 Hz, J = 2.2 Hz). 13C NMR (400 MHz-methanol-d6) d: 166.05 (C-2); 103.41(C-3); 183.49 (C-4); 163.05 (C-5); 95.91(C-6); 166.05 (C-7); 101.31 (C-8); 159.72 (C-9); 104.27 (C-10); 123.43 (C-1′); 113.89 (C-2′); 147.31 (C-3′); 151.65 (C-4′); 116.86 (C-5′); 120.23 (C-6′).

**(2)** Apigenin 1H NMR (400 MHz, Methanol-d6) d: 6.55 (s, 1H, H-3); 6.16 (d, 1H, H-6, J = 2.0 Hz); 6.39 (d, 1H, H-8, J = 2.0 Hz); 7.35 (d, 1H, H-2′ and H-6′, J = 2.2 Hz); 6.88 (d, 1H, H-3′ and H-5′, J = 8.2 Hz); 13C NMR (400 MHz-Methanol-d6) d: 170.32 (C-2); 103.60 (C-3); 183.69 (C-4); 163.15 (C-5); 100.93 (C-6); 166.08 (C-7); 95.65 (C-8); 163.93 (C-9); 104.65 (C-10); 123.25 (C-1′); 129.42 (C-2′ and C-6′); 117.14 (C-3′ and C-5′); 159.65 (C-4′).

### High-Performance Liquid Chromatograph Coupled With a Diode-array UV-Vis Detector

Chromatographic analyses were performed on HPLC-DAD-UV using a Shimadzu Nexera XR^®^ liquid chromatographer coupled to a Shimadzu UV detector with diode array SPDM20A, equipped with a CBM20A controller, DGU20A degasser, LC20AD binary pump, CTO20A oven, and SILA20A auto-injector. A Shimadzu LabSolutions Software Version 5.3 (Shimadzu, Kyoto, Japan) was used to analyze chromatograms. Combinations of acidified ultrapure water (pH 3.0, with anhydrous acetic acid, Merck, Darmstadt, Germany) (A) and acetonitrile (HPLC grade, Tedia, Rio de Janeiro, Brazil) (B) were used as the mobile phase (initially 5% A rising to 95% in 80 min). HPLC column was silica-based C18 (250 mm × 4.6 mm i.d. × 5 μm particle size, ODS Hypersil, Thermo, Waltham, MA, United States). The oven was set at 50°C and the injection volume was 10 μL for all analyses.

### Preparation of *A. chica* Hydroethanolic Extract (ACCE) and Flavone-Rich Fraction (ACFF) Samples

A total of 1,000 µL of acetonitrile: methanol (both HPLC grade, Tedia, Rio de Janeiro, Brazil) mixture (75:25; v/v) was added to 5 mg of ACCE and ACFF, previously weighed in a 4 mL vial. The vial was sealed and the sample was sonicated for 10 min with occasional swirling. Posteriorly, the sample was vortexed to mix thoroughly, followed by filtering through a 0.45 µm PTFE filter (Merck Millipore, Darmstadt, Germany) before further analyses into an HPLC vial.

### Preparation of Standard Solutions and Quantification of Luteolin and Apigenin

Stock solutions of analytical standards luteolin and apigenin (Lot. 2,578 and 2,968, Phytolab, Vestenbergsgreuth, Germany) were prepared at 1,000 mg/mL in MeOH (Tedia, Rio de Janeiro, Brazil) in volumetric flasks. Six concentration of work solutions (1; 4; 8; 12; 16, and 20 μg/mL) were done on the day for calibration curves of each compound. The solutions were filtered in a 0.45 μm PTFE filter (Merck Millipore, Darmstadt, Germany) before analyses by HPLC-DAD-UV. Injections of 20 μL were performed in triplicate to obtain the calibration curves from the areas corresponding to the peaks of luteolin and apigenin. The analytical curve (1–20 μg/mL) of the standards was plotted based on the UV-Vis signal at 254 nm: luteolin content (μg/mL) = (Abs (mAu) + 21,030)/83,557; R2 = 0.9995 and apigenin content (μg/mL) = (Abs (mAu) + 27,059)/77,296; R2 = 0.996. Flavones amounts were calculated in mg/g of dry extract. The following dilution factors were used for luteolin and apigenin quantitative analysis: 147.06 and 11.47, respectively.

### Liquid Chromatography Coupled to Electrospray ionization Mass spectrometry in Tandem Analysis

Liquid chromatography coupled to electrospray ionization mass spectrometry in tandem (LC-ESI-MS-MS) was performed with an LC Shimadzu Nexera Ultra-Fast Liquid Chromatography (UFLC) coupled to an ion trap Bruker amaZon MS. Analyses were performed at ambient temperature in a silica-based C18 column (150 mm × 4.6 mm i.d. x 2.6 μm particle size, Kinetex C18 gravity column, Phenomenex, CA, United States). The mobile phase consisted of ultrapure water obtained from the Milli-Q Millipore purification system, acidified at pH 3.0 with anhydrous acetic acid (Merck, Darmstadt, Germany) (A) and acetonitrile (HPLC grade, Tedia, Rio de Janeiro, Brazil) (B). The gradient of B was as follows: in 54.86 min from 5 to 95%; from 54.86 to 55.54 min returns to 5% B, remaining like this until 62.0 min to column re-equilibration. The flow rate was set at 0.5 mL/min and the injection volume was 1 μL. ESI-MS/MS were recorded in a Bruker Ion trap amazon SL mass spectrometer in the positive ionization mode (ESI+). The operating conditions were 1 μL/min infusion, 3.0–4.0 kV capillary voltage, 100°C temperature source, and cone voltage of 20–40 V. Mass spectra were recorded and interpreted by Bruker Compass Data Analysis 4.2 (Bruker Daltonics, Boston, MA, United States).

### Animals and Ethical Statements

All procedures performed with 4–6-weeks old female BALB/c mice were in accordance with the National Council for Control of Animal Experimentation (CONCEA). These animals were obtained from the Institute of Science and Technology in Biomodels of Oswaldo Cruz Institute and the experiments were approved by the local Ethics Committee on Animal Care and Utilization (CEUA-IOC L53/2016).

### Parasites

*Leishmania amazonensis* H21 (MHOM/BR/76/MA-76) was maintained in the laboratory by successive passages in BALB/c mice. Parasites were isolated from a non-ulcerated nodular lesion in the footpad and amastigote viability was checked by light microscopy. 10^6^ amastigote forms were transferred to the NNN medium (Novy-MacNeal-Nicolle) and maintained for seven days to differentiate into promastigote forms. Then, these forms were cultured at 26°C in Schneider’s Insect medium (Sigma, St Louis, MO, United States), supplemented with 10% fetal bovine serum (Gibco, Gaithersburg, MD, United States), 100 IU/mL of penicillin, and 100 μg/mL of streptomycin in a maximum of ten *in vitro* passages (Almeida-Souza et al., 2016).

### Peritoneal Macrophage Obtaining and Cell Culture

The animals were previously inoculated intraperitoneally, with 3.0 mL of 3% sodium thioglycolate. After 72 h of stimulation, the animals were euthanized with 10% ketamine and 2% xylazine according to the weight of each animal and, after death, the abdomen skin was retracted for peritoneum exposure. 10.0 mL of sterile pH 7.2 phosphate-buffered saline was inoculated and a light manual massage was performed. The cells were harvested from the peritoneum with the same syringe and dispensed in a sterile conical tube to prepare the cell suspension. The cells were centrifuged at 2,000 rpm for 5 min and suspended in RPMI 1640 medium supplemented with 10% fetal bovine serum (FBS), penicillin (100 U/mL) and (100 μg/mL) streptomycin, at 37°C and 5% CO_2_ and grown overnight (Almeida-Souza et al., 2018).

### Activity Against *L. amazonensis* Promastigote Forms

To evaluate the anti-promastigote effects of the ACCE, ACFF, and isolated flavonoids from *A. chica* on the promastigote forms of *L. amazonensis*, viable promastigotes were counted in a Neubauer chamber according to the method described by Rottini et al. (2019). In brief, 50 µL of the promastigotes (2 × 10^6^ cells/mL) harvested from the logarithmic growth phase were added to 96-well flat-bottomed microtiter plates. Then, 50 µL of the ACCE (62.5–1,000 μg/mL), ACFF (12.5–200 μg/mL) or isolated flavonoids (3.125–100 μg/mL) were added to each well and incubated at 26 ± 1°C for 72 h. Wells with parasites and DMSO 1% only were used as untreated control and amphotericin B (0.03125–1.0 μg/mL) was used as a reference drug. After the incubation, viable promastigotes were counted in a Neubauer chamber. The experiments were conducted in triplicate. Percentage of growth inhibition was calculated from the count of viable parasites relative to the untreated control, and 50% inhibitory concentration (IC_50_) values were determined.

### Cytotoxicity Assay

Peritoneal macrophages collected as previously described (*Peritoneal Macrophage Obtaining and Cell Culture*) were seeded at 5 × 10^5^ cells per milliliter in a 96-well plate and allowed to adhere overnight at 37°C and 5% CO_2_. Subsequently, the cells were treated with different concentrations of the ACCE, ACFF (both with concentrations of 7.81–1,000 μg/mL), flavonoids (1.95–500 μg/mL) or amphotericin B (0.19–25 μg/mL), in a final volume of 100 μL/well, incubated for 72 h at the same conditions. Wells without cells were used as blank and wells with cells and 1% DMSO were used as controls. The cytotoxicity was determined with the MTT (Sigma, St Louis, MO, United States) assay. Half-maximal cytotoxic concentration (CC_50_) was calculated according to Oliveira et al. (2018).

### Activity Against *L. amazonensis* Intracellular Amastigotes and Selectivity Index

Peritoneal macrophages of BALB/c mice were cultured in 24-well plates (5 × 10^5^ cells/well), containing round coverslips and incubated at 37°C in 5% CO_2_ overnight. The cells were then infected with promastigote forms of *L. amazonensis*, in the parasite/cell ratio of 10:1, for 6 h followed by washing with PBS to remove free parasites. Infected cells were treated with different concentrations of ACFF (0.15–2.5 μg/mL) or luteolin (1.56–25 μg/mL), and the plates were incubated in similar conditions for 24 h. The macrophages containing amastigotes without compounds treatment and those treated with amphotericin B (2.5–0.15 μg/mL) were considered the negative and positive controls, respectively. Finally, the coverslips of the infected and treated cells were fixed in Bouin's solution, stained by Giemsa and observed under a light microscope. The activity anti-intramacrophage amastigotes of the compounds was evaluated by counting the number of amastigotes in each macrophage by examining 200 macrophages in comparison with the untreated control. The percentage of infected cells was obtained from the number of infected cells divided by two. The mean number of amastigotes per cell was obtained from the number of intracellular amastigotes in 200 cells divided by the number of infected cells (Almeida-Souza et al., 2020). The percentage inhibition was calculated and IC_50_ was obtained by GraphPad Prism^®^ version 7 (GraphPad Software Inc., San Diego, CA, United States). Selectivity index (SI), which was calculated using the equation of CC_50_ for murine macrophage/IC_50_ for the intracellular amastigote forms of *L. amazonensis*, was used to compare the toxicity and activity of the compounds.

### Nitrite Quantification

Nitric oxide (NO) release was indirectly measured in the supernatants of macrophage culture (5 × 10^6^ cells/mL) by the Griess reaction for nitrite according to the method described by Almeida-Souza et al. (2016). About 50 μL of the supernatants were collected 48 h after treatment with ACFF (2.5 μg/mL) or luteolin (25 μg/mL) and/or stimulation twith *L. amazonensis* (3 × 10^7^ parasites/mL), and added in 96-well plates. Then, were added to supernatants 50 µL of Griess reagent (25 µL of sulfanilamide 1% in 2.5% H_3_PO_4_ solution and 25 µL of N-(1-naphthyl ethylenediamine 0.1% solution). After 10 min, the plates were read at 570 nm on the spectrophotometer and the nitrite values were obtained from the standard sodium nitrite curve (1.5–100 μM).

### Transmission Electron Microscopy

Promastigote forms of *L. amazonensis* were treated with IC_50_ for ACFF or luteolin, for 24 h. Non-treated parasites were used as a negative control. After 24 h-incubation at 26°C promastigotes were collected by centrifugation at 5,000 rpm for 5 min. The parasites were fixed with 2.5% glutaraldehyde (Sigma, St Louis, MO, United States) in 0.1 M sodium cacodylate buffer, pH 7.2, overnight. Then, parasites were washed three times with 0.1 M sodium cacodylate buffer and post-fixed in a solution containing 1% osmium tetroxide, 0.8% potassium ferrocyanide, and 5 mM calcium chloride, washed in 0.1 M sodium cacodylate buffer, dehydrated in graded acetone, and embedded in EPON 812 resin (Sigma, St Louis, MO, United States). Ultrathin sections were obtained from 100 nm cuts in Sorvall MT 2-B (Porter Blum) ultramicrotome (Sorvall, Newtown, CT, United States) stained with 5% uranyl acetate aqueous solution and lead citrate (1.33% lead nitrate and 1.76% sodium citrate), and examined in a transmission electron microscope JEM-1011 (JEOL, Tokyo, Japan) operating at 80 kV (Mondêgo-Oliveira et al., 2021).

### Statistical Analysis

The numerical results were expressed as mean ± standard deviation and the statistical analyses were conducted through the statistical software GraphPad Prism^®^ version 7. The differences were considered significant when *p* < 0.05 by one-way analysis of variance (ANOVA) and Mann-Whitney test.

## Results

### High-Performance Liquid Chromatograph Coupled With a Diode-Array UV-Vis Detector and Liquid Chromatography Coupled to Electrospray Ionization Mass Spectrometry in Tandem

**FIGURE 1:**
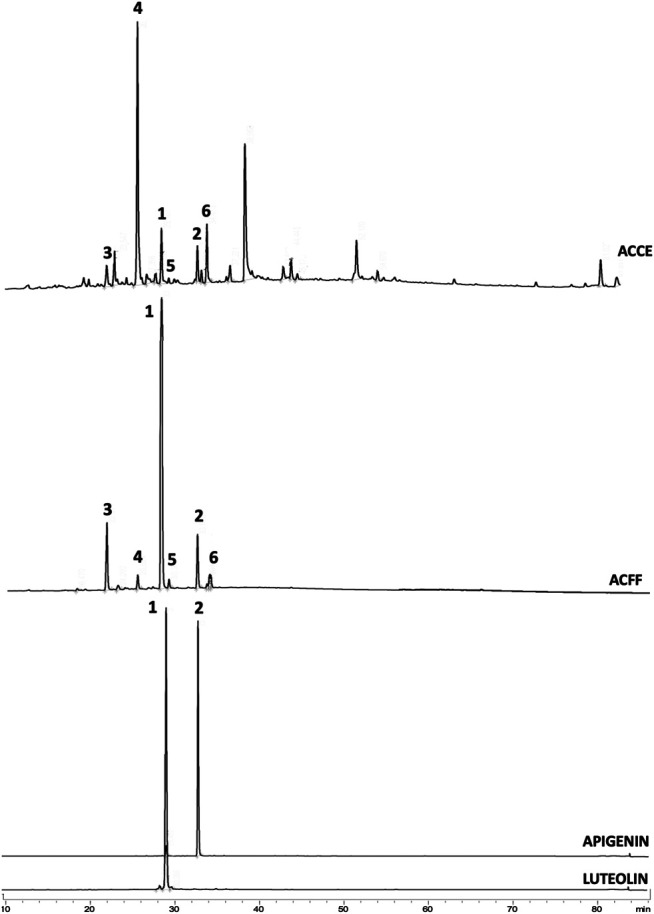
Chromatograms obtained from HPLC-DAD-UV analysis of **(A)**. *Arrabidaea chica* crude extract (ACCE) showing all peaks including non-flavone compounds; **(B)**. *A. chica* flavone-rich fraction (ACFF) showing 6 peaks identified as flavones: luteolin (**1**), apigenin (**2**), 6-hydroxy-luteolin (**3**), scutellarein (**4**), carajuflavone (**5**) and chrysoeriol (**6**); **(C)**. luteolin (Rt = 29.34 min); **(D)**. Apigenin (Rt = 33.67 min).

The comparison between the profiles of ACCF and ACFF of *A. chica* (Figure 1) by HPLC-DAD-UV showed that the chromatographic fractionation steps were useful both to obtain a fraction rich in flavones, and isolating two of them, luteolin **(1)** and apigenin **(2)**, which had already been reported in the literature, and had their structures confirmed by ^1^H and ^13^C NMR (Özgen et al., 2011; Siraichi et al., 2013).

**FIGURE 2:**
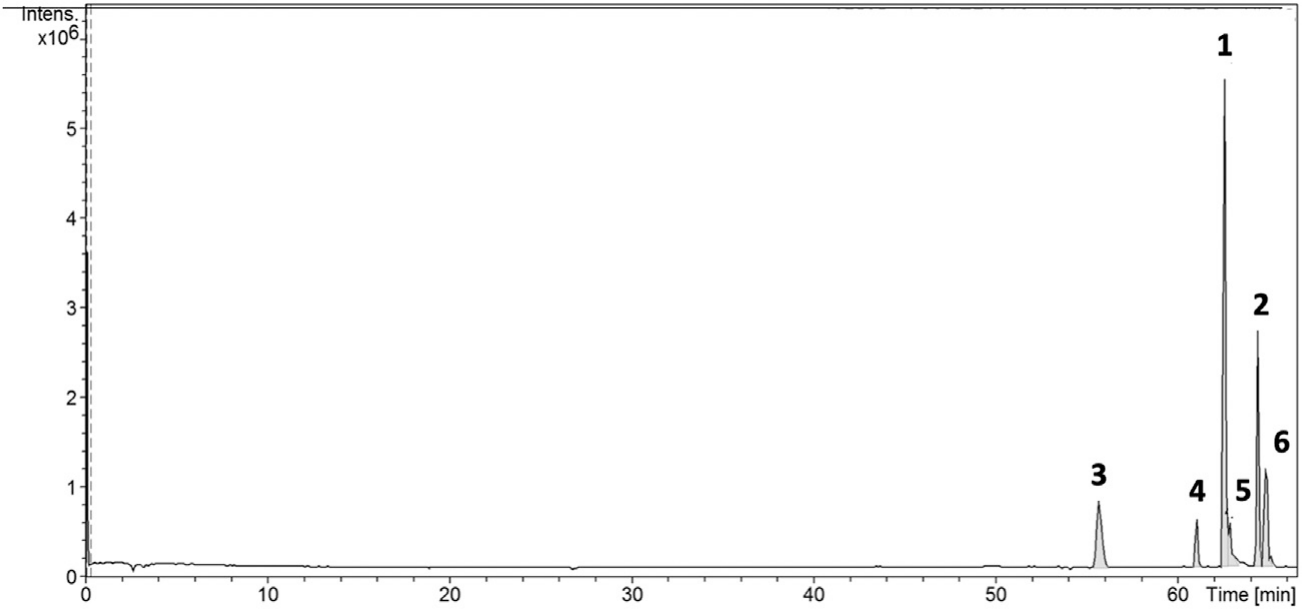
Chromatogram obtained from LC-ESI-MS-MS analysis of the flavone-rich fraction of *Arrabidaea chica* Verlot, also presenting 6 peaks of flavones. Luteolin (**1**), apigenin (**2**), 6-hydroxy-luteolin (**3**), scutellarein (**4**), carajuflavone (**5**), and chrysoeriol (**6**).

LC-ESI-MS-MS analysis in positive mode of the flavone-rich fraction (ACFF) also resulted in a chromatogram with six peaks (Figure 2). Mass spectrometry in tandem (MS-MS) and pseudomolecular ions [M + H]^+^ were useful to identify and confirm the structures of the six flavones in flavone-rich fraction.

The results of HPLC-DAD-UV and LC-ESI-MS-MS allowed the identification of the six component flavones of the rich fraction in flavonoids of *A. chica*, as shown in Table 1.

**TABLE 1:** LC-ESI-MS-MS and HPLC-DAD-UV data of flavone-rich fraction obtained from *Arrabidaea chica* Verlot.

Peak	LC-ESI-MS-MS			HPLC-DAD-UV		Identification
	Rt (min)	[M + H]	MS-MS	Rt (min)	UV data (nm)	
3	55.7	303.0451	303, 257, 169	22.9	346, 282	6-OH-luteolin
4	61.0	287.0496	287, 169	26.6	338, 283	Scutellarein
1	62.6	287.0493	287, 241, 153	29.8	348, 256	Luteolin
5	62.9	317.0594	317, 302, 168	30.3	346, 271	Carajuflavone
2	64.4	271.0548	271, 153	33.6	339, 267	Apigenin
6	64.8	301.0464	301, 286, 258,153	35.0	343, 267	Chrysoeriol

LC-ESI-MS-MS: Liquid chromatography coupled to electrospray ionization mass spectrometry. HPLC-DAD-UV: High-performance liquid chromatography coupled to a diode-array detector. [M + H]: pseudomolecular ions. MS-MS: Mass spectrometry. Rt: retention time.

### Quantification of Luteolin and Apigenin in the Flavone-Rich Fraction

Chromatographic investigation of the analytes was realized by comparing the retention time (Rt) and UV spectra of the corresponding peaks in the flavone-rich fraction with authentic standards apigenin and luteolin. Flavones quantification was carried out through calibration curves obtained by triplicate injections. Calibration curves showed to be linear in the ranges of 1–20 μg/mL for luteolin and apigenin. Besides that, good regression coefficients (*r* ^2^) for linear regression equations of both standards were obtained: 0.9995 for luteolin and 0.9960 for apigenin. Luteolin and apigenin contents were calculated using the equations Absorbance (mAu) = 83,557 (concentration)—21,030 and Absorbance (mAu) = 77,296 (concentration)—27,059, respectively. The content of luteolin and apigenin (Table 2) were obtained in mg/g of dry weight, after correction by dilution factor, when necessary.

**TABLE 2:** Quantification of luteolin and apigenin (mg/g dry extract) content in the flavone-rich fraction of *Arrabidea chica* Verlot.

Flavone	Area (mAU)	Concentration (mg/g)	RSD (%)
Luteolin (**1**)	1,062,597 ± 1,326.92	190.717 ± 0.015	0.12
Apigenin (**2**)	811051.3 ± 4,500.54	12.4367 ± 0.058	0.54

Values are expressed as the mean ± SD (*n* = 3, see experimental). RSD: relative standard deviation; Content (mg/g) for luteolin = (area +21,030)/83,557*147.06 (dilution factor); content (mg/g) for apigenin = (area +27,059)/77,296*11.47 (dilution factor).

### Leishmanicidal Activity of Flavonoids Obtained From *A. chica*

Growth inhibitory activity by the selected compounds was performed on *L. amazonensis* promastigotes forms. In the test, all evaluated compounds from *A. chica* showed antipromastigote effects (Table 3). The results also revealed that ACFF caused leishmanicidal effects on the promastigotes of *L. amazonensis* 3-fold more potent in comparison with the ACCE. Moreover, the IC_50_ value for the flavonoids apigenin and luteolin against promastigotes of *L. amazonensis* were similar to their original fraction, ACFF.

**TABLE 3:** Cytotoxicity and antileishmanial activity of extracts and isolated flavonoids from *Arrabidaea chica*.

Compounds	Cytotoxicity	*L. amazonensis*		
	CC_50_ (μg/mL)	Promastigote	Intracellular amastigote	
		IC_50_ (μg/mL)	IC_50_ (μg/mL)	SI
ACCE	38.64 ± 1.23	121.8 ± 1.41	—	—
ACFF	40.93 ± 1.18	40.42 ± 0.10	3.575 ± 1.13	11.44
Apigenin	11.87 ± 1.32 (43.92 μM)	45.60 ± 1.08 (168.7 μM)	—	—
Luteolin	8.005 ± 1.23 (27.97 μM)	31.51 ± 1.13 (110.08 μM)	11.78 ± 1.24 (41.15 μM)	0.679
Amphotericin B	9.352 ± 1.11 (10.1 μM)	0.07198 ± 1.15 (0.078 μM)	0.2752 ± 1.28 (0.298 μM)	33.98

ACCE: *A. chica* crude extract; ACFF: fraction rich in flavonoids of *A. chica*. Data represent mean ± SD of at least two experiments realized in triplicate. CC_50_: half-maximal cytotoxic concentration for 50% of cells; IC_50_: 50% inhibitory concentration of parasites; SI: selectivity index, SI = CC_50_/IC_50_ intracellular amastigote.

Evaluation of cytotoxicity showed that ACFF was the less cytotoxic compound, followed by ACCE, and the flavonoids apigenin and luteolin were more cytotoxic having CC_50_ similar to amphotericin B (Table 3). The cytotoxicity against peritoneal macrophage and *L. amazonensis* were compared using the selectivity index (SI) (Table 3).

From the results described in Table 3, ACFF and luteolin were selected for evaluation against intra-macrophage forms. We found that luteolin inhibited the intracellular amastigote number. However, results demonstrated that the ACFF was 3.3-fold more effective for the amastigotes than luteolin. Therefore, considering this increased activity against intracellular amastigote, we observed a promising selectivity for the fraction rich in flavonoids (SI/24 h > 10.0). Amphotericin B showed leishmanicidal activity and cytotoxicity as expected.

**FIGURE 3:**
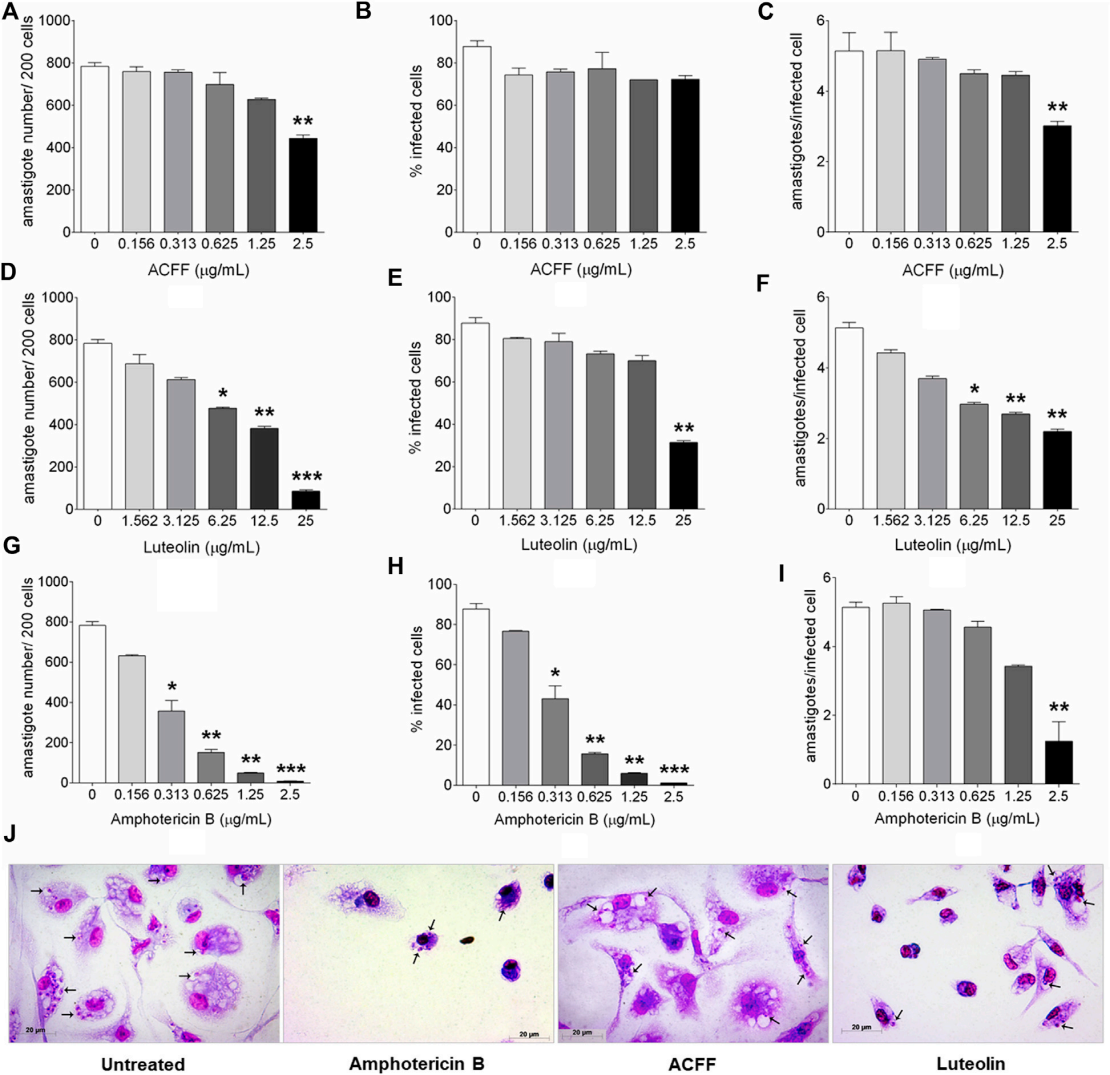
BALB/c peritoneal macrophages infected with *Leishmania amazonensis* and treated for 24 h with amphotericin B, *Arrabidaea chica* flavone-rich fraction (ACFF) or luteolin. **(A–I)** Parameters of infection and **(J)** light microscopy of untreated, and treated with amphotericin B (2.5 μg/mL), ACFF (2.5 μg/mL) or luteolin (25 μg/mL) infected cells. (black arrows) Intracellular amastigotes inside macrophages. The images and data (mean ± standard deviation) represent two independent experiments performed in triplicate. *p < 0.05, **p < 0.01 and ***p < 0.001 when compared with untreated infected cells by Kruskal–Wallis and Dunn’s multiple comparison test. Giemsa, 40× objective.

The parameters of infection of untreated cells were used as comparative control for the treatment with the different compounds at different concentrations. Infected and untreated macrophages presented 783.1 ± 80.43 amastigotes per 200 cells, 87.74 ± 7.95% of infected cells, and mean of amastigotes per infected cell of 5.135 ± 0.53 (Figure 3). The treatment with ACFF significantly reduced the number of amastigotes per 200 cells (443.50 ± 30.60, *p* = 0.0010, Figure 3A) and the mean of amastigotes per infected cell (3.19 ± 0.21, *p* = 0.0043, Figure 3C) at 2.5 μg/mL. In infected cells treated with luteolin, a statistically significant reduction was observed at the highest concentration evaluated (25 μg/mL) in all parameters of infection according to the intracellular amastigote number (84.50 ± 13.28, *p* = 0.0002, Figure 3D), percentage of infected cells (31.50 ± 1.73, *p* = 0.0011, Figure 3E), and the mean of amastigotes per infected cell (2.20 ± 0.10, *p* = 0.0049, Figure 3F). Amphotericin B showed a statistically significant reduction in all infection parameters at 2.5 μg/mL (9.00 ± 1.73, *p* = 0.0005, Figure 3G; 1.09 ± 0.21, *p* = 0.0002, Figure 3H; 1.25 ± 1.37, *p* = 0.0002, Figure 3I). As shown in Figure 3J, images of BALB/c peritoneal macrophages infected with *L. amazonensis* and treated with amphotericin B, ACFF and luteolin corroborate with the results of Figures 3A–I.

### Nitrite Quantification in *L. amazonensis*-Infected Peritoneal Macrophages Treated With ACFF and Luteolin

**FIGURE 4:**
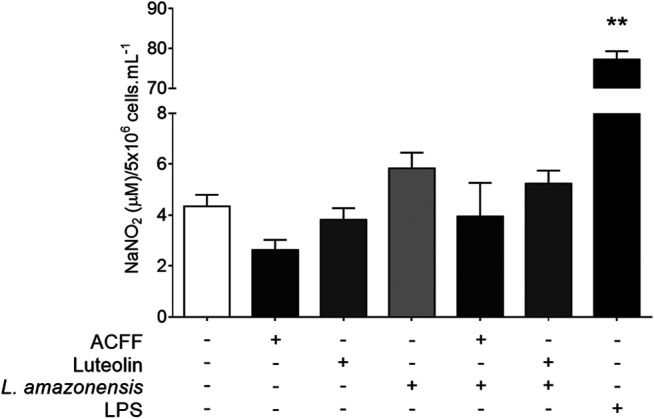
Nitrite quantification in the supernatant of BALB/c peritoneal macrophages treated with *Arrabidaea chica* flavone-rich fraction (ACFF) (2.5 μg/mL) or luteolin (25 μg/mL), stimulated or not with *Leishmania amazonensis*. Data represents mean ± standard deviation of experiment realized in sextuplicate; **p < 0.01 when compared with untreated and unstimulated macrophages by Mann-Whitney test.

The effect of *A. chica* compounds on nitrite production in the supernatant of BALB/c peritoneal macrophages is shown in Figure 4. The macrophages showed low nitrite levels in cells treated with ACFF (2.63 ± 0.79 μM NaNO_2_, *p* = 0.0857) and luteolin (3.55 ± 0.91 μM NaNO_2_, *p* = 0.4000), when compared to untreated cells (4.34 ± 1.12 μM NaNO_2_). Low nitrite levels were also observed in cells stimulated with *L. amazonensis* and treated with ACFF (3.34 ± 2.65 μM NaNO_2_, *p* = 0.4476) and luteolin (4.43 ± 0.30 μM NaNO_2_, *p* = 0.4048), when compared to stimulated and untreated cells (5.83 ± 1.69 μM NaNO_2_), although the difference was not statistically significant. In the test, macrophages when stimulated with lipopolysaccharide (LPS) produced high levels of nitrite compared to cultures not stimulated with LPS.

### Flavones From *A. chica* Promotes Ultrastructural Changes in *L. amazonensis* Promastigotes

**FIGURE 5:**
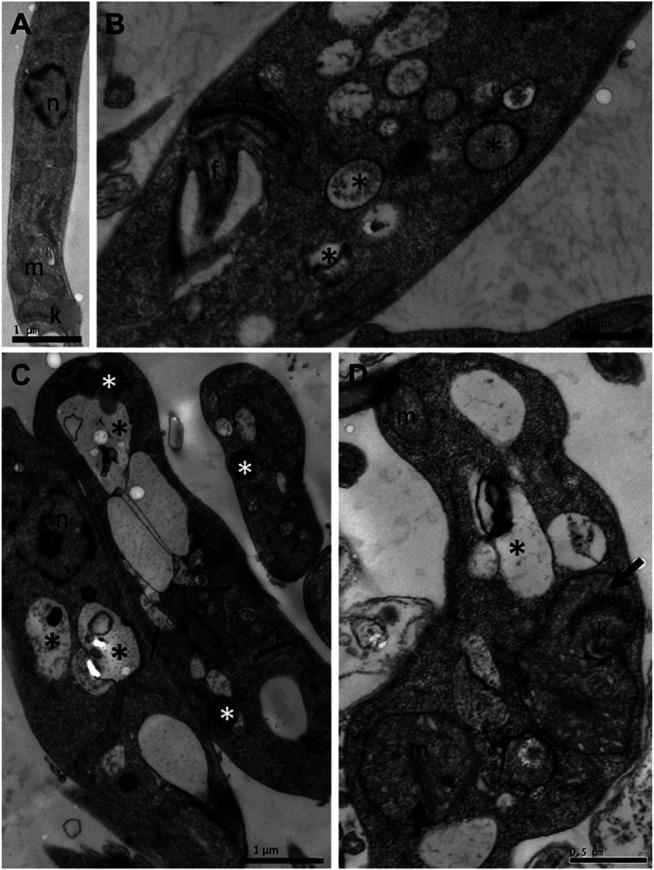
Transmission electron microscopy of *Leishmania amazonensis* promastigote forms. **(A)** Untreated parasites. **(B–D)** Parasites were treated with *Arrabidaea chica* flavone-rich fraction (ACFF) at 2.5 μg/mL for 24 h. Several vacuoles containing granular, circular, and electron-dense material dispersed by the cytoplasm (black asterisks); lipid bodies; multivesicular bodies (thin arrows); swelling of kinetoplast and mitochondria with breakdown of mitochondrial cristae (thick arrow). n: nucleus; m: mitochondria; k: kinetoplast; f: flagellum.

**FIGURE 6:**
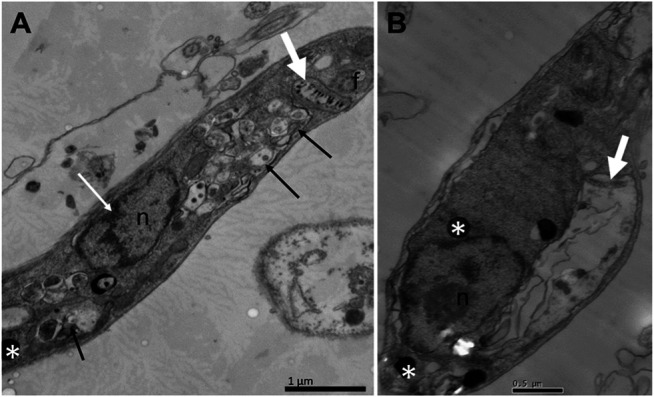
Ultrastructural alterations of *Leishmania amazonensis* promastigote forms treated with luteolin at 25 μg/mL for 24 h. Small vacuoles containing electron-dense microvesicles dispersed by the cytoplasm (arrows), change in the nuclear chromatin (white arrow) **(A)**; lipid bodies (white asterisks), kinetoplast and mitochondria fully degenerated (white thick arrows) **(A–B)**. n: nucleus; f: flagellum.

**FIGURE 7:**
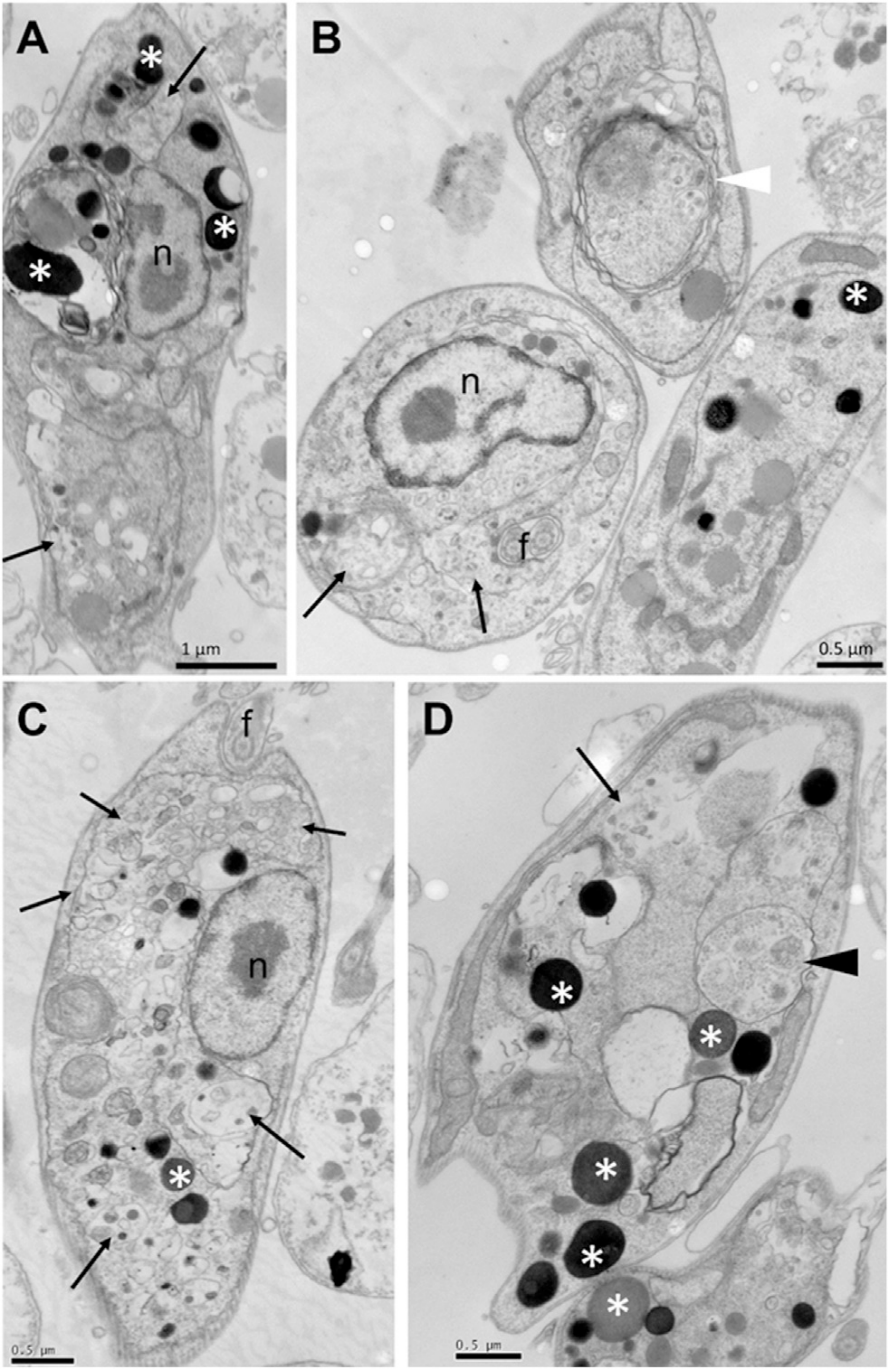
Ultrastructural alterations of *Leishmania amazonensis* promastigote forms treated with luteolin at 25 μg/mL for 24 h. Vacuoles containing material of different shapes and density (arrows), lipid bodies (white asterisks) **(A–D)**, several layers of circular membranes involving multivesicular bodies (white arrowhead) **(B)**, autophagosome-like vacuoles (black arrowhead) **(D)**. n: nucleus.

To assess whether the treatment with compounds from *A. chica* promoted morphological and structural changes, analysis by transmission electron microscopy (TEM) was performed. Parasites were treated or not with ACFF and luteolin IC_50_ for 24 h. Promastigote forms showed the cellular morphology with an elongated body and all its intact organelles (Figure 5A). It was possible to observe that the treatment with ACFF promoted several vacuoles containing granular, circular and electron-dense material dispersed by the cytoplasm (Figure 5B), lipid bodies, multivesicular bodies (Figure 5C), swelling of kinetoplast and mitochondria with the breakdown of mitochondrial cristae (Figure 5D). Luteolin promoted small vacuoles containing electron-dense microvesicles dispersed in the cytoplasm (Figures 6A,B), change in the nuclear chromatin (Figure 6A), lipid bodies (Figures 6A,B), kinetoplast and mitochondria fully degenerated (Figures 6A,B). Also, it was possible to observe that luteolin promoted vacuoles containing material of different shapes and density, lipid bodies (Figures 7A–D), several layers of circular membranes involving multivesicular bodies (Figure 7B), and autophagosome-like vacuoles (Figure 7D).

## Discussion

Dichloromethane fraction obtained from hydroalcoholic extract of *A. chica* is admittedly rich in flavonoids (Takemura et al., 1995; Zorn et al., 2001; Devia et al., 2002; Barbosa et al., 2008; Siraichi et al., 2013), among which stand out anthocyanidins, the chemical markers of the species, in addition to flavones and flavonols. The fractionation carried out in this study had as the main objective obtaining a flavone-rich fraction, without anthocyanidins, and the isolation of some major compounds.

HPLC-DAD-UV analysis showed a chromatogram with six peaks. UV/Vis spectrum showed that the six compounds had broad bands of absorption with maximum peaks in the range of 338 and 348 nm (band I) and 256 and 283 nm (band II). Band I is associated with absorption due to the B ring of cinnamoyl system and when registered in the range of 304–350 nm it is characteristic of flavones (Mabry et al., 1970). Band II is related to absorption in the A ring benzoyl system. This ring when trisubstituted shows higher absorption at band II (Supplementary material—S1).

The flavone-rich fraction (ACFF) was also analyzed by LC-ESI-MS-MS, whose results compared with literature data (Siraichi et al., 2013; Grabsk et al., 2017) allowed to identify four flavonoids: 6-hydroxy-luteolin **(3)**, scutellarein **(4)**, carajuflavone **(5)** and chrysoeriol **(6)**.

Mass spectrometry has been an extremely useful method to identify and characterize polyphenolic secondary plant metabolites, and flavonoids are of particular importance (Cuyckens and Claeys, 2004). The collision of the precursor ions produced in the positive ion mode from the detected flavones yielded mass spectra for product ions. These spectra were used for their structural identification. The most useful fragmentations in terms of flavonoid aglycone identification are those that require cleavage of two carbon-carbon bonds of the flavonoid C ring (dihydropyran ring), which can be rationalized in terms of retro-Diels-Alder reactions (Pinheiro and Justino, 2012; Vaca et al., 2017). Such fragmentation in general allows a quick identification of the flavonoid type, as well as the number of substituents in each ring (Pinheiro and Justino, 2012). Therefore, in positive mode, fragments at *m/z* 153 and 169 are characteristic of compounds containing di-hydroxylated and tri-hydroxylated A ring, respectively. Retro-Diels-Alder fragmentation also produces information about B ring. Fragments at *m/z* 119 and 135 are related to mono- and di-hydroxylated B ring, respectively. Positive LC-ESI-MS-MS data analyses were performed to confirm structures in addition to data already obtained in HPLC-DAD-UV and Rt of flavones from ACFF. Both luteolin **(1)** and apigenin **(2)** have been identified by ^1^H NMR and ^13^C NMR spectrometric techniques, UV, elution order, and Rt, and were also evaluated by fragmentation pattern. These compounds showed the Retro-Diels-Alder fragmentation pattern confirming the di-hydroxylation of A ring (ions at *m/z* 153.0151 and 153.0148, respectively). Luteolin **(1)** showed experimental pseudo-molecular ion (M + [H]^+^) at *m/z* 287.0496, which is compatible to the molecular formula C_15_H_10_O_6_ as well as the mass fragment at *m/z* 241.0447 [(M-H_2_O-CO)]^+^. Apigenin **(2)** was confirmed by the pseudo-molecular ion at *m/z* 271.0548, corresponding to the molecular formula C_15_H_10_O_5_, as well as the fragments at *m/z* 243 characterized by C=O loss. Retro-Diels-Alder fragments correspondent to a tri-hydroxylated A ring at *m/z* 169.0103 and 169.0105 were observed to 6-hydroxy-luteolin **(3)** and scutellarein **(4)**, respectively. The compound 6-hydroxy-luteolin or 5,6,7,3′,4′-penta-hydroxy flavone **(3)** also showed the experimental pseudo-molecular ion (M + [H]^+^) at *m/z* 303.0449, compatible to the molecular formula C_15_H_11_O_7_, as well as the mass fragment at *m/z* 257.0389 [(M-H_2_O-CO)]^+^, confirming their identification. The structure of scutellarein **(4)** was also confirmed by the experimental pseudo-molecular ion at *m/z* 287.0496, which is compatible with the molecular formula C_15_H_10_O_6_. Carajuflavone or 6,7,3′,4′-tetrahydroxy-5-methoxy flavone **(5)** showed a pseudo-molecular ion at *m/z* 317.0595 corresponding to the molecular formula C_16_H_13_O_7_. The fragment at *m/z* 168.0017 indicated that in Retro-Diels-Alder fragmentation, one of the three hydroxyl groups of A ring was substituted. It was confirmed by their fragmentation pattern that produced ions at *m/z* 302.0360 confirming the loss of a methyl group (M—[CH_3_]^+^). Chrysoeriol **(6)** showed experimental pseudo-molecular ion at *m/z* 301.0646 compatible with the molecular formula C_16_H_13_O_6_. Chrysoeriol fragmentation pattern produced ions at *m/z* 286.0414 [(M)-CH_3_]^+^ and at *m/z* 258.0467 [(M)-CH_3_-CO]^+^. These pieces of evidence confirm compound **(6)** as chrysoeriol (Supplementary Material S2).

All identified flavones in ACFF have been previously described for *A. chica*. Compound 6-hydroxy-luteolin, besides of luteolin, apigenin, and chrysoeriol had been described for *A. chica* leaves hydroethanolic extract (Vasconcelos et al., 2019). In another study, also with an *A. chica* extract, Siraichi et al. (2013) identified six flavones, from which, four—6-hydroxyluteolin **(3)**, luteolin **(1)**, apigenin **(2)**, and scutellarein **(4)**—were identified in ACFF. Carajuflavone **(5)**, another 6-hydroxylated compound from *A. chica* had already been described by Takemura et al. (1995). Compound 6-hydroxy-luteolin **(3)** has been reported in the Bignoniaceae family, and 6-hydroxylation is a common structural feature of the Bignoniaceae flavonoids having particular chemotaxonomy relevance for this reason. Besides, 6-hydroxylation is also characteristic of the structures of the red pigments anthocyanidins carajurin and carajurone found in *A. chica* (Harborne, 1967).

Quantitative analysis showed luteolin **(1)** as the most representative flavone of ACFF (Figure 1), whose concentration was calculated as 190.7 mg/g dry extract. Apigenin **(2)** represented only 12.43 mg/g dry extract. The results obtained in the present study are already better than other publications on concentrations and contents of luteolin and apigenin in extracts obtained from *A. chica* leaves (Paula et al., 2013). It demonstrated that the developed method for obtaining the flavone-rich fraction showed great results.

Publications by our research network previously reported on the leishmanicidal activity of hydroalcoholic extracts from four morphotypes of *A. chica* (Moragas-Tellis et al., 2020). However, as the species *A. chica* exhibit a great set of polyphenolic compounds—mainly flavonoids—we decided to expand this study, previously done only with anthocyanidins, to evaluate whether flavones may also be involved in the leishmanicidal activity.

The increase in antipromastigote activity from bioguided fractionation facilitated the identification of the best fractionation stage that contributes to the best results for leishmanicidal activity. As a result, the ACFF was more effective than ACCE, and the isolated flavone luteolin showed better activity than ACFF. Apigenin, however, showed activity similar to ACFF.

Studies conducted by Cortez de Sá et al. (2015) presented a screening test for the crude ethanolic extract of the leaves of *A. chica* with the inhibitory concentration of 50% (IC_50_) of the promastigote forms of *L. amazonensis* determined at 155.9 μg/mL. Phytochemical screening of that study showed an extract containing flavonoids, phenolic compounds, tannins, anthocyanidins and chalcones. In addition, the crude extract of the leaves of *A. chica* had a cytotoxic effect at a concentration of 189.9 μg/mL. Factors that can explain the difference in phytochemical and biological results are probably due to the climatic differences between the two locations where the plant was collected, as well as the way of obtaining the extract (Cortez de Sá et al., 2015). However, it should be noted that the difference in cytotoxicity found with our study is probably related to the exposure time used, since we used 72 h, while in the study by Cortez de Sá et al. (2015) the time was reduced to 24 h. Besides, and even more important, the different morphotypes of *A. chica* vary in chemical composition and, consequently, in biological activity (Moragas-Tellis et al., 2020).

Aware that the extraction and fractionation methods can help in biological activity, we opted to optimize the bioguided fractionation process favoring the obtaining of a flavone-rich fraction in an attempt to better explore the phytochemical profile of *A. chica* species. Flavones have demonstrated a wide range of biological activities that include antioxidant, antimicrobial, anti-inflammatory and other activities. In addition, structure-activity relationships have generated interest among medicinal chemists, making the flavones an important class of natural products of new therapeutic agents (Singh et al., 2014).

Literature data show flavones with leishmancidal activity. Apigenin was tested for its anti-*Leishmania* activity against the promastigote forms of *L. amazonensis*, inhibiting the growth of the parasites at IC_50_ values of 23.7 μM (Fonseca-Silva et al., 2015) and 22.77 μM against *Leishmania donovani* strains (Antwi et al., 2019). This inhibition was also observed by luteolin at IC_50_ value of 12.5 µM against *L. donovani* (Mittra et al., 2000). Another study shows apigenin and luteolin having inhibitory activity against *L. donovani* axenic amastigotes (IC_50_ 1.9 and 0.8 μg/mL, respectively) (Tasdemir et al., 2006). The antipromastigote activity of the ACFF may be related to the high concentration of these flavones in their composition. The high cytotoxicity of these flavones against L6 cells (derived from rat skeletal myoblasts) has also been reported, at IC_50_ values of 18.1 μg/mL for apigenin and 9.44 μg/mL for luteolin—data that corroborate the toxicity observed in our study. Besides, the structure-activity relationship of these flavones was investigated, and it was observed that the presence of the 5,7-dihydroxybenzochromone structure greatly increases leishmanicidal activity, occurring the same with the presence of the double bond between positions 2 and 3 (C-2,3). Such characteristics are observed in apigenin and luteolin. Thus, it is possible to infer that the different leishmanicidal activity of these two flavones might be due to the replacement pattern in the B-ring, since luteolin has a catechol portion (3′,4′-dihydroxyphenyl) while apigenin has only one hydroxyl in 4' (Tasdemir et al., 2006).

Our findings against the promastigote form recommend selecting the ACFF and luteolin, the main component of the fraction—to verify the activity against the forms of *L. amazonesis* intracelular amastigote. In the search for new drugs against *Leishmania* spp., intracellular amastigote is the stage of the parasite considered as the most relevant target for the primary screening of new compounds (de Muylder et al., 2011), and the most consistent indicator of *in vivo* activity (Croft, 1986), additionally considered "the gold standard" of in *in vitro* studies (Baek et al., 2020). It is therefore of interest to test the effectiveness of *A. chica* compounds in intracellular amastigotes.

It was possible to observe an improvement in the leishmancidal activity, being ACFF and luteolin 11.3 and 2.67 times more active against intracellular amastigote forms than to the promastigotes forms, respectively. A study performed by Wong et al. (2012) reported that luteolin exhibited promising activity only against the intracellular amastigote, but not for extracellular promastigotes, suggesting that its specific targets are present only in the intracellular phase. This activity is also observed in another study against *L. donovani*, according to which luteolin reduced intracellular amastigote load by 70% at a final concentration as low as 12.5 μM (Mittra et al., 2000). Therefore, the high concentration of luteolin in ACFF may be responsible for the increased inhibition against the intracellular forms of the parasite.

When comparing the results of the activity against the intracellular phase, ACFF was 3.2 times more active than luteolin. It is worth mentioning that another flavone present in the ACFF composition was apigenin, but this compound was not tested against the intracellular amastigote form due to its low yield. However, data from the literature show the significant inhibitory effect of apigenin against the intracellular amastigote forms of *L. donovani*, at IC_50_ values of 45.66 ± 0.01 μM (12.34 μg/mL). In addition, when the infected macrophages were treated with increasing concentrations of apigenin, there was a decrease in the number of infected cells (Antwi et al., 2019). Therefore, this leads us to infer that the presence of a couple of compounds in the ACFF can contribute to the leishmanicidal activity and to lower cytotoxic effect, favoring the greater selectivity to the parasite observed to ACFF when compared to luteolin. These findings are often considered to be the result of a synergistic or additive effect of the extract's constituents (Dalby-Brown et al., 2005).

The inhibition of intracellular amastigote is directly related to the presence of nitric oxide (NO) in activated macrophages (Mukbel et al., 2007). Therefore, in an attempt to understand the leishmanicidal activity of ACFF and luteolin against intracellular amastigote forms, nitrite quantification was performed as an indirect way to determine NO levels (Almeida-Souza et al., 2016). However, there were no significant changes in this assessment parameter.

In a study with apigenin and luteolin, both inhibited NO production, considering that a C-2,3 double bond may be important, and that the patterns of substitution of flavonoid molecules can determine the potency of the inhibition in NO production (Kim et al., 1999). In addition, suppression in the production of NO and prostaglandin E2 (PGE2), without having cytotoxicity in RAW 264.7 mouse macrophage cells activated by bacterial lipopolysaccharide was also observed when exposed to flavones, luteolin and its luteolin-7-O-glucoside. The suppression of inducible nitric oxide synthase (iNOS) and the expression of cyclooxygenase-2 protein (COX-2) are responsible for the inhibitory effects, and not for the reduction of enzymatic activity (Hu and Kitts, 2004). Different culture conditions and cell types may also be responsible for some difference between data in the literature and the results of the present study. Therefore, other mechanisms may be involved in the leishmanicidal activity of *A. chica* flavonoids against intracellular amastigote.

Trying to understand the leishmanicidal effect of *A. chica* compounds directly on the parasite, ultrastructural evaluation of the promastigote forms of *L. amazonensis* was performed by transmission electron microscopy. The treatment with ACFF promoted ultrastructural alterations such as the vacuolization process of the cytoplasm, lipid and multivesicular bodies, swelling of the kinetoplast and mitochondria with the breaking of the mitochondrial ridges. Ultrastructural changes were also observed in the study by Rodrigues et al. (2014), with *L. infantum* promastigotes treated with fraction B2 (1:1 n-hexane/ethyl acetate) obtained from the crude hexane extract of *A. chica*. In this study, mitochondrial edema with loss of matrix content and the presence of vesicles within this organelle were observed.

Luteolin, the metabolite with the highest concentration in ACFF composition, was evaluated for ultrastructural alterations induced in *L. amazonensis* promastigotes. It was possible to observe vacuolization of the cytoplasm, change in nuclear chromatin, lipid bodies, with kinetoplast and fully degenerated mitochondria, and vacuoles similar to autophagosomes. Studies performed by Mittra et al. (2000) indicates that flavonoids may target the enzyme topoisomerase II in the kinetoplast of parasites, since it reports that luteolin and quercetin induce significant cleavage of the topoisomerase II-mediated kDNA minicircle in *Leishmania*, an inhibition similar to the well-known anti-*Leishmania* drug, pentamidine. Another study elucidates the mechanism of action of luteolin by analyzing mitochondrial and cytosolic changes associated with death similar to *L. donovani* cell apoptosis (Sen et al., 2006). In this work, Sen et al. (2006) reports that luteolin inhibition of the production of glycolytic ATP was an essential event responsible for the depolarization of the mitochondrial membrane in depleted mt-DNA cells to propagate apoptosis-like death in *Leishmania* cells.

Our results provide additional evidence on the antileishmanial activity induced by *A. chica*. However, based on the findings obtained, further studies should be carried out to elucidate the mechanisms of action, as well as *in vivo* studies are essential to assess the leishmanicidal activity of the ACFF against *Leishmania*.

## Conclusion

Chromatographic techniques and analysis of the mass spectra obtained from the ACCE allowed the identification of compounds of the ACFF derived from *A. chica*. The flavones luteolin and apigenin were isolated from the ACFF using chromatographic techniques and identified by NMR spectrometry techniques. ACCE, ACFF and flavones showed leishmanicidal activity against the promastigotes of *L. amazonensis*. The antiparasitic effect of ACFF and luteolin was confirmed by the ultrastructural changes with induction of mitochondrial damage. ACFF also showed low cytotoxicity in host cells if compared with the isolated flavones. ACFF and luteolin showed leishmanicidal activity against the intracellular amastigotes, however, this activity is not related to the production of NO by host cells. Thus, ACFF is a suitable candidate for further *in vivo* investigations against *L. amazonensis*.

## Data Availability

All datasets presented in this study are included in the article/Supplementary Material.
