# Supplementary material for: Antileishmanial Activity of Flavones-Rich Fraction From Arrabidaea chica Verlot (Bignoniaceae)
Source: Front Pharmacol. 2021 Jul 20;12:703985. doi: 10.3389/fphar.2021.703985 (PMC8329660; doi:10.3389/fphar.2021.703985)
Supplement: Supplementary file 1 [file DataSheet1.PDF]

Supplementary Material

Antileishmanial activity of flavones-rich fraction from *Arrabidaea chica* Verlot (Bignoniaceae)

João Victor Silva-Silva, Carla Junqueira Moragas-Tellis, Maria do Socorro dos Santos Chagas, Paulo Victor Ramos de Souza, Celeste da Silva Freitas de Souza, Daiana de Jesus Hardoim, Noemi Nosomi Taniwaki, Davyson de Lima Moreira, Maria Dutra Behrens, Kátia da Silva Calabrese*, Fernando Almeida-Souza

* Correspondence: calabrese@ioc.fiocruz.br

Supplementary Figures

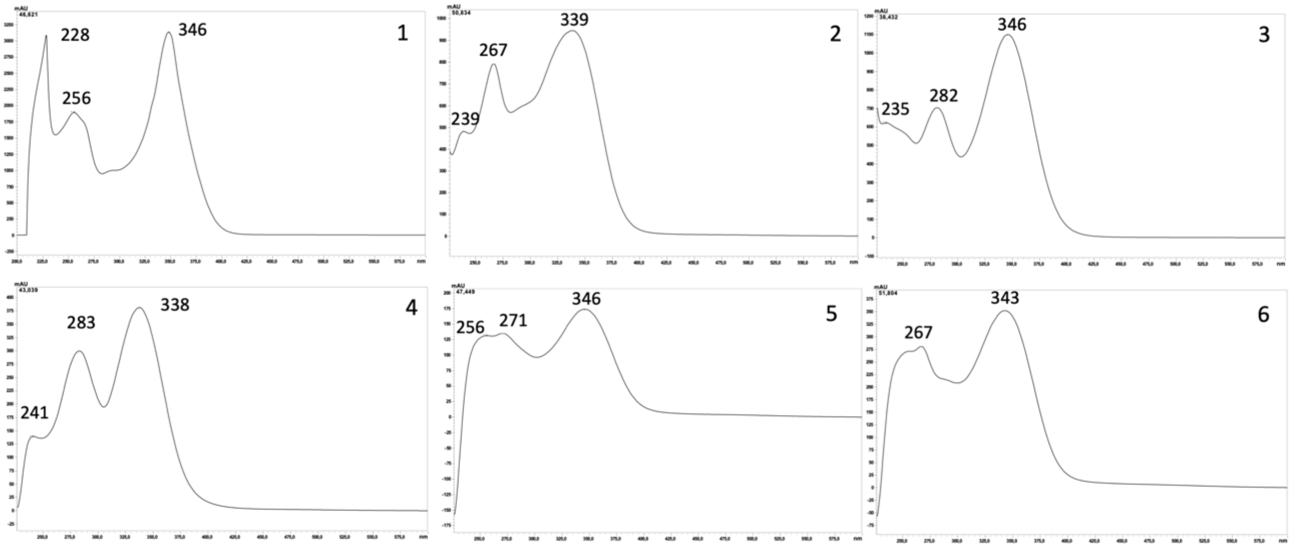

Supplementary Figure 1. UV Spectral data of six flavones identified at the flavone rich fraction from *Arrabidaea chica*: **(1)** luteolin; **(2)** Apigenin; **(3)** 6-Hydroxy-luteolin; **(4)** Scutellarein; **(5)** Carajuflavone and **(6)** Chrysoeriol.

1. Luteolin

Molecular formula $C_{15}H_{10}O_6$

$[M+H]^+$ experimental: 287.0496; $[M+H]^+$ calculated: 287.0447; Δ = -18.81 ppm

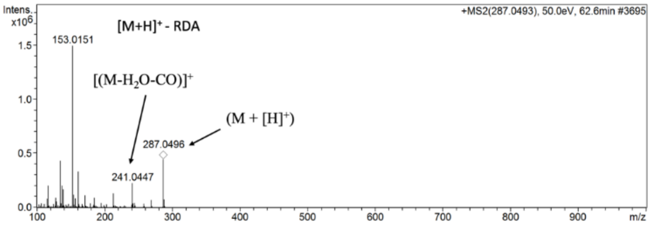

1. Mass spectra of luteolin showing signals (M + [H]$^+$) at 287.0496 ; $[(M-H_2O-CO)]^+$ at 241.0447 and $[M+H]^+$ - Retro dials alder fragmente at 153.0151 ppm.

2. Apigenin

Molecular formula $C_{15}H_{10}O_5$

$[M+H]^+$ experimental: 271.0548; $[M+H]^+$ calculated: 271.0601; Δ = -19.55 ppm

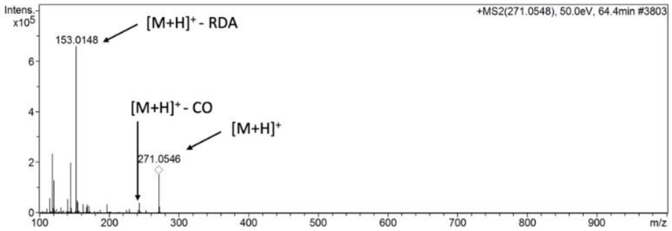

Mass spectra of apigenin showing signals (M + [H]$^+$) at 271.0548 ; $[M+H]^+$ - CO at 243 and $[M+H]^+$ - Retro dials alder fragment at 153.0148 ppm.

3. 6-hydroxy-luteolin

Molecular formula $C_{15}H_{11}O_7$

$[M+H]^+$ experimental: 303.0449; $[M+H]^+$ calculated: 303.0499; Δ = -16.49 ppm

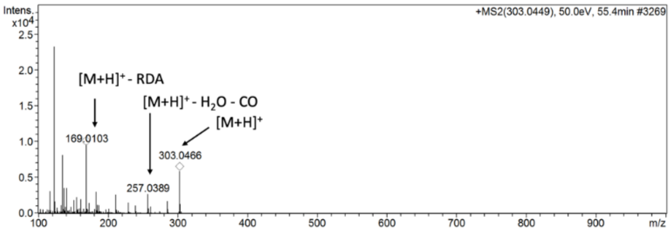

Mass spectra of 6-hydroxy-luteolin showing signals (M + [H]$^+$) at 303.0466 ; $[M+H]^+$ - H_2O - CO at 257.0389 and $[M+H]^+$ - Retro dials alder (RDA) fragment at 169.0103 ppm.

4. Scutellarein

Molecular formula $C_{15}H_{10}O_6$

$[M+H]^+$ experimental: 287.0496; $[M+H]^+$ calculated: 287.0550; Δ = -18.86 ppm

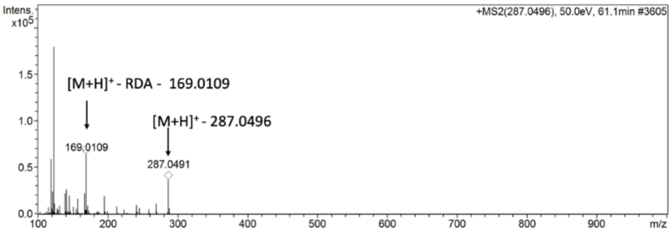

Mass spectra of scutellarein showing signals (M + [H]$^+$) at 287.0496 and $[M+H]^+$ - Retro dials alder (RDA) fragment at 169.0109 ppm.

5. Carajuflavone

Molecular formula $C_{16}H_{13}O_7$

$[M+H]^+$ experimental: 317.0595; $[M+H]^+$ calculated: 317.0655 ; Δ = -18.92 ppm

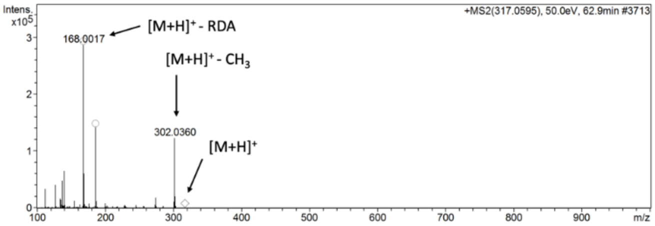

Mass spectra of carajuflavone showing signals (M + [H]$^+$) at 317.0595; $[M+H]^+$ - CH_3 at 302.0360 and $[M+H]^+$ - Retro dials alder (RDA) fragment at 168.0017 ppm.

6. Chrysoeriol

Molecular formula $C_{16}H_{13}O_6$

$[M+H]^+$ experimental: 301.0646; $[M+H]^+$ calculated: 301.0706 ; Δ = -20.14 ppm

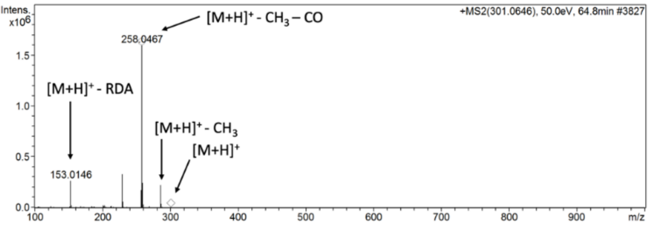

Mass spectra of carajuflavone showing signals (M + [H]$^+$) at 301.0646; $[M+H]^+$ - CH_3 at 286.0414; $[M+H]^+$ - CH_3 – CO at 258.0467 and $[M+H]^+$ - Retro dials alder (RDA) fragment at 153.0146 ppm.

Supplementary Figure 2. Mass spectral data of six flavones identified at the flavone rich fraction from *Arrabidaee chica*: (1) luteolin; (2) Apigenin; (3) 6-Hydroxy-luteolin; (4) Scutellarein; (5) Carajuflavone and (6) Chrysoeriol.
